# A Web-Based Stratified Stepped Care Mental Health Platform (TourHeart): Semistructured Interviews With Stakeholders

**DOI:** 10.2196/35057

**Published:** 2022-05-13

**Authors:** Emily W S Tsoi, Winnie W S Mak, Connie Y Y Ho, Gladys T Y Yeung

**Affiliations:** 1 Department of Psychology, The Chinese University of Hong Kong Shatin, NT China (Hong Kong); 2 New Life Psychiatric Rehabilitation Association Kowloon China (Hong Kong)

**Keywords:** stepped care, recovery, engagement, eHealth, mental health, mental well-being, psychological intervention

## Abstract

**Background:**

TourHeart, a web-based stratified stepped care mental health platform, is a one-stop solution that integrates psychoeducation and other well-being promotional tools for mental health promotion and mental illness prevention and evidence-based, low-intensity psychological interventions for the treatment of people with anxiety and depressive symptoms. Instead of focusing only on symptom reduction, the platform aims to be person-centered and recovery-oriented, and continual feedback from stakeholders is sought. Understanding the perspectives of users and service providers enables platform developers to fine-tune both the design and content of the services for enhanced service personalization and personal recovery.

**Objective:**

This qualitative study evaluated a web-based mental health platform by incorporating the perspectives of both users and service providers who administered the platform and provided coaching services. The platform included both web-based and offline services targeting adults along the mental health spectrum based on the two-continua model of mental health and mental illness.

**Methods:**

Interview questions were designed based on the Reach, Effectiveness, Adoption, Implementation, and Maintenance framework (RE-AIM). Views on offline services, the design of the web-based platform, user experience, and the contents of the platform were explored using semistructured interviews. A total of 27 service users and 22 service providers were recruited using purposive criterion sampling. A hybrid thematic analysis was performed to identify salient aspects of users’ and providers’ experiences with and views of the platform.

**Results:**

Totally, 3 broad themes (namely, the quality of the platform, drivers for platform use, and coaching services) emerged from the interview data that highlighted users’ views of and experiences with the web-based platform. The platform’s general esthetics, operations, and contents were found to be critical features and drivers for continued use. Although coaching services were indispensable, participants preferred the autonomy and anonymity associated with web-based mental health services.

**Conclusions:**

This study highlights the importance of web-based mental health services being easy to navigate and understand, being user-centric, and providing adequate guidance in self-help. It also confirms existing design standards and recommendations and suggests that more rigorous, iterative user experience research and robust evaluation should be conducted in the future adaptation of web-based stratified stepped care services, so that they can be more personalized and better promote personal recovery.

## Introduction

### Background

This study aimed to evaluate a web-based stratified stepped care mental health platform for the working adult population. Under the stratified stepped care approach, the platform recommends services that are commensurate with the intensity needed by users based on the results of a brief self-report mental health assessment on anxiety, depression, and flourishing that was completed at the beginning and then provides timely step-down or step-up options to users upon completion of interventions at the recommended level and based on their latest mental health status. This approach aims to optimize the provision of services based on users’ needs and efficient use of resources, given that the mental health of the working adult population is often overlooked. Specifically, mental ill-health is a tremendous burden on the society owing to loss of productivity and quality of life among the workforce. A meta-analysis [1] on the global prevalence of common mental disorders in the general adult population (aged 16-65 years) indicated a global common mental disorders prevalence of 17.5% (in the past 12 months). The statistics vary across geographical locations. Among all high-income countries, individuals from English-speaking countries (eg, United States and United Kingdom) reported the highest prevalence (19%), followed by European countries (17.1%) and Asian countries (11.5%). A report from the World Health Organization in 2017 revealed that the total number of people living with depression is 322 million worldwide, and nearly half of these people live in the South-East Asia region and Western Pacific region [2]. Despite the substantial social and economic impact of mental ill-health and its associated loss of health and functioning on individuals and the society, help seeking, especially in Asian countries, remains low. Commonly cited barriers to help seeking include stigma, long waiting time, high costs, and concerns about privacy and anonymity [3,4].

In addition to mental ill-health, whether the general population is flourishing is also an important concern, as it impacts the extent to which individuals can attain complete mental health [5]. Under the two-continua model of mental health and mental illness, any person can fall within the orthogonal quadrants, from languishing to flourishing in the dimension of mental health and from no mental illness to having mental illness in the dimension of mental illness [6]. Thus, in addition to paying attention to the prevention and treatment of mental illness, promotion of mental health is equally essential at the population level to maximize public mental health and personal recovery [7].

Given the growing concerns about the accessibility, affordability, scalability, and anonymity of mental health promotion and help seeking for mental illness, digital mental health apps and platforms have burgeoned as robust solutions in recent years. A review of the literature [8] identified several digital mental health service types, including psychoeducation, screening and assessment, social support, guided self-help, and intervention.

For digital interventions, evidence has been established regarding their effectiveness in promoting well-being, reducing mental illness and stigma around help seeking, and ameliorating stress and psychological distress [9-11]. Typically, digital interventions are designed based on a single treatment approach such as mindfulness-based training [12,13], cognitive behavioral therapy (CBT) [14,15], or a combination of multiple treatment approaches [16], although increasing amount of research has suggested transdiagnostic approaches to be effective in tackling underlying mechanisms for common mental disorders such as anxiety and depression [17].

Although stratified care can provide timely services to individuals that are commensurate with their mental health needs, stepped care service begins with the least restrictive care to individuals to maximize resources so that users with varying levels of needs are provided the same intensity of services and may progress to services of high intensity on an as-needed basis. Its accessibility and flexibility in stepping up and down are particularly suitable for promoting mental health and reducing mental illness within the same web-based platform for a population with varying levels of flourishing, anxiety, and depression. Through mobile technology, different levels of services can be tailored and delivered to individuals who would otherwise not pay attention to their mental health or receive timely services, as their conditions are usually considered as less urgent and less acute than others with more severe mental health needs. People with low levels of mental health needs are also found to be more hesitant in seeking help for their issues [18].

### TourHeart—Web-Based Stratified Stepped Care Platform

**Figure 1:**
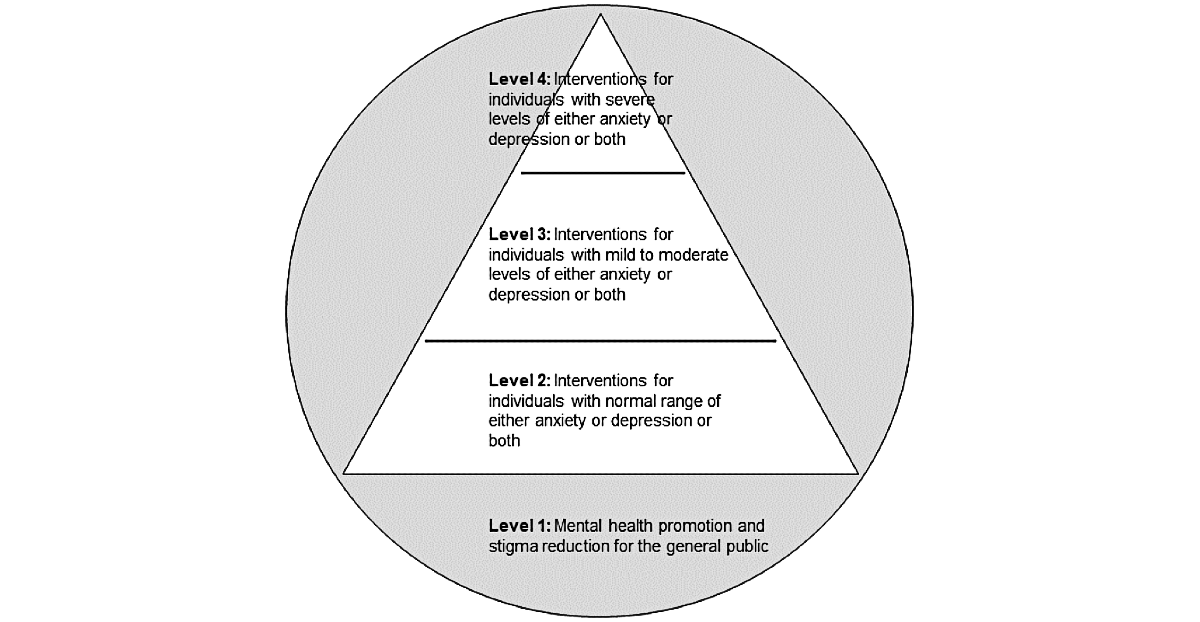
Stratified stepped care model for mental well-being.

This study aimed to evaluate a stratified stepped care service platform with both offline and web-based components (The Jockey Club TourHeart Project) that was designed based on the matched care and stepped care principles [19,20]. The web-based platform targets adults on a spectrum, from flourishing with normal to severe range of anxiety and depressive symptoms. Through a simple sign-in procedure, users are directed to a web-based personal assessment in which their mental health profiles are obtained. At the time of writing, 9738 individuals have registered, signed in, and completed the battery of mental health profiles at least once. On the basis of their initial scores for flourishing (assessed by the Flourishing Scale) [21], anxiety symptoms (assessed by the 7-item Generalized Anxiety Disorder questionnaire) [22], and depressive symptoms (assessed by the 9-item Patient Health Questionnaire) [23,24], users are recommended services that are commensurate with their current mental health state based on 4 levels (see Table 1 for the service-level criteria). Level 1 targets the general public and includes social media and public events such as psychoeducational talks and public exhibitions that promote mental health awareness and reduce mental illness stigma (ie, offline events). Level 2 provides self-guided web-based mental well-being promotion training programs for improving well-being and reducing psychological distress for those who present with mild levels of anxiety or depressive symptoms across the flourishing spectrum. Web-based courses for this level include stress management, emotion regulation training, self-compassion training, and mindfulness-based training. Each training course consists of 4 modules, and participants are invited to complete 1 module each week sequentially. Level 3 provides coach-guided web-based psychological interventions for alleviation of anxiety and depressive symptoms based on guided self-help principles for those who present with moderate levels of anxiety and depressive symptoms across the flourishing spectrum. At this level, users can access mindfulness-based intervention or rumination-focused CBT (RFCBT) [25]. These interventions contain 6 sequential modules, with each module requiring 1 week of commitment to complete. Users are guided at the end of each module by feedback on their written homework and exercises, through email from well-being coaches (psychology graduates with specific training in low-intensity psychological interventions). Finally, level-4 services are low-intensity psychological interventions for those who present with moderately severe to severe levels of anxiety and depressive symptoms across the flourishing spectrum. Participants at this level receive individual sessions of low-intensity CBT (LiCBT) delivered by trained psychological well-being officers in person or via videoconferencing. Figure 1 shows the stratified stepped care model for mental well-being used in TourHeart. Owing to the complexity and fluidity of the platform where multiple components may interact with users’ experiences and thus affect users’ outcomes [26], qualitative method is particularly useful in encapsulating the perspectives of multiple stakeholders. Therefore, service users and service providers (event organizers, project administrators, and well-being coaches) at all levels were invited to participate in this study to provide their views on the platform.

**Table 1:** Service levels and criteria^a^.

Level	PHQ-9^b^ score, range	Combination logic	GAD-7^c^ score, range	Combination logic	Suicide risk (PHQ-9—score for question 9=2 to 3)
1	N/A^d^	N/A	N/A	N/A	N/A
2 (nonclinical to mild)	0-9	AND	0-7	AND	No
3 (moderate)	10-14	OR	8-14	AND	No
4 (moderately severe to severe)	15-27	OR	15-21	OR	Yes
5 (complex mental health issues)	—^e^	—	—	—	—

^a^Flourishing is assessed for all platform users. Levels 2 to 4 are reserved for individuals who have registered on the platform, whereas level 1 is targeted to all working populations.

^b^PHQ-9: 9-item Patient Health Questionnaire.

^c^GAD-7: 7-item Generalized Anxiety Disorder questionnaire.

^d^N/A: not applicable.

^e^Not available (manually assigned and referred out).

### Aims and Objectives

Given the importance of understanding the contents and salient features of the platform that may exert positive effects on its users to improve future design and user experience, this study aimed to explore the views and preferences of users and service providers regarding the implementation of the TourHeart platform with both offline and web-based services targeted for working adults along the spectra of flourishing, anxiety, and depressive symptoms. The specific study objectives were to (1) explore users’ views on the design and quality of web-based and offline mental health services and (2) identify drivers for web-based platform use.

## Methods

This qualitative study used semistructured interviews that were conducted via phone or videoconferencing with both users and service providers to explore the experiences and perceived usefulness of the services and the platform. The technical and design aspects of the web-based platform were also explored.

### Ethics Approval

This study obtained ethics approval from the corresponding author’s (WWSM) home institution (Clinical Research Ethics Committee 2018.654) and followed the Standards for Reporting Qualitative Research [27].

### Recruitment

To maximize the variation in experiences obtained from our participants, purposive criterion sampling [28] was used. A list of potential participants was generated from the back end of the platform. Participants who have accessed TourHeart for any of the 4 levels or have taken courses from levels 2 to 4 in the past 4 weeks from the time of the study were identified as potential participants. The target sample size was 6 participants from level 1, 8 participants each from levels 2 and 3, and 6 participants from level 4. Half of the participants from levels 2 to 4 (10/21, 48%) were *completers* and the remaining participants were *dropouts*, defined as those who attempted >30% but <100% of the course.

Participants were recruited on a rolling basis until the target sample size was achieved. For the user group, potential participants were invited via WhatsApp messages and a follow-up phone call to explain the purpose and implications of joining the research in detail. A total of 58 participants were invited, of whom 21 (36%) declined or did not respond. Then, the consenting participants (27/58, 47%) were invited to complete a consent form via the web, where their basic demographic information was also collected. After obtaining consent, phone interviews were scheduled. Each interview lasted for an average of 40 minutes.

The service provider group (n=22) consisted of both past and current research, administrative, and service staff. They were invited either in person or via WhatsApp messages. Consenting staff were invited to complete a web-based consent form, through which basic demographic information was also collected. To increase synergy between study participants and to obtain detailed responses, separate focus groups were conducted based on the roles of the participants. The grouping of roles included (1) project administrators (project manager and project administrative assistant), (2) past research staff, (3) current research staff and well-being coaches, and (4) volunteers who organize offline events. Each group consisted of 2 staff members.

All participants, except the current project staff, received a HK $100 (US $12.75) honorarium in the form of cash as a token of appreciation for the time spent for the study.

### Interviews

The interview protocol was designed based on the RE-AIM (Reach, Effectiveness, Adoption, Implementation, and Maintenance) framework [29], focusing on areas that were most relevant to the participants’ needs and experiences with the web-based platform. In the users’ version, aspects related to reach, effectiveness, and implementation were explored using open-ended questions. In the service providers’ version, aspects related to reach, effectiveness, adoption, service implementation, and future maintenance were explored. Equipped with previous knowledge and experiences with the web-based platform, the interviewer used probes where appropriate; digressions were allowed for obtaining organic responses. All conversations were recorded and transcribed verbatim.

### Data Analysis

An initial codebook was developed by the 2 interviewers using a hybrid approach (inductive and deductive), followed by thematic analytic procedure [30]. Thematic analysis typically consists of 6 phases. They include (1) familiarizing with the raw data, (2) initial coding, (3) searching for themes, (4) reviewing the themes, (5) defining and naming themes, and (6) reporting of findings. These 6 phases do not have to be followed in a sequential manner. To ensure data integrity, triangulating questions were asked during all the interviews to confirm the researcher’s own understanding of the participants’ views; in addition, notes were taken during each interview. Moreover, immediately after each interview, brief analytic summaries were made to document the researcher’s personal observations, such as tone of the participant’s voice. These steps were taken to ensure that reporting remained as faithful to the original narratives as possible. NVivo (version 12; QSR International) was used to conduct the analysis.

## Results

### Participants

Most participants in the user group (n=27) were women (22/27, 82%), with a mean age of 34 (SD 10.11; range 19-58) years. Most of these participants (16/27, 59%) reported to have obtained at least tertiary-level education. Participants came from diverse work backgrounds. One-third of the participants were employed in the education sector (9/27, 33%), followed by those employed in public health and welfare sector (4/27, 15%), banking (3/27, 11%), and public services (3/27, 11%). The remaining participants were from other industries. Regarding the positions held, of the 70% (19/27) of the participants who opted to provide information about their work, 47% (9/19) participants reported to be holding executive positions and an equal number of the remaining participants reported to be office workers (5/19, 26%) or homemakers and carers (5/19, 26%) at the time of the interview. Finally, most of those from the service provider group were women (18/22, 82%), with a mean age of 34 (SD 10.27; range 21-46) years.

### Overview of Key Findings

**Figure 2:**
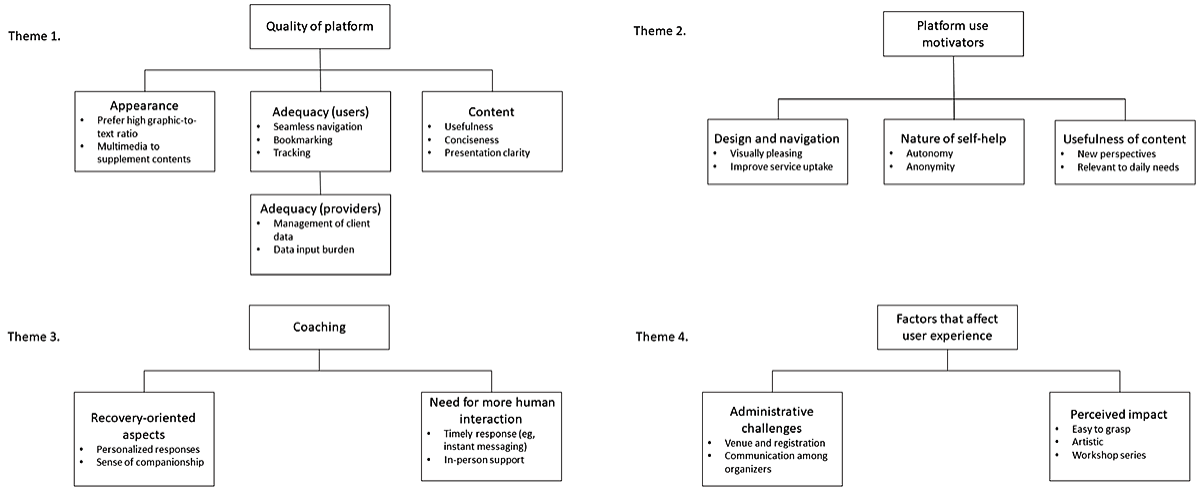
Themes and subthemes.

This study focused on evaluating both the offline and web-based services offered by the TourHeart platform. For evaluation of offline services, two subthemes emerged from one broad theme (*factors that influence users* *experience*), which were (1) administrative challenges and (2) perceived impact. For the web-based services, findings pertinent to the positive and desired features of the platform that can inform future development and implementation were analyzed and reported. With this analytical goal in mind, codes from 21 user interviews and 14 service provider interviews were grouped into three major themes: (1) quality of the platform, (2) motivating factors for platform use, and (3) comments on human coaching. These themes were divided into subthemes. The themes and an overview of associated findings are summarized in Figure 2.

### Web-Based Services

#### Theme 1: Quality of the Platform

##### Overview

This part of the study involved 60% (21/35) users (level 2: 8/21, 38%; level 3: 8/21, 38%; and level 4: 5/21, 24%) and 40% (14/35) providers. At the beginning of the interview, participants were invited to freely share their experience of using the platform to identify critical platform design parameters perceived by its users. Responses from this segment of interviews were coded into 3 main subthemes, namely, appearance, adequacy, and content of the platform.

##### Appearance

In this subtheme, comments on the graphics, graphics-text balance, use of multimedia, style consistency, word length, language and style, and color choices were collated.

**Figure 3:**
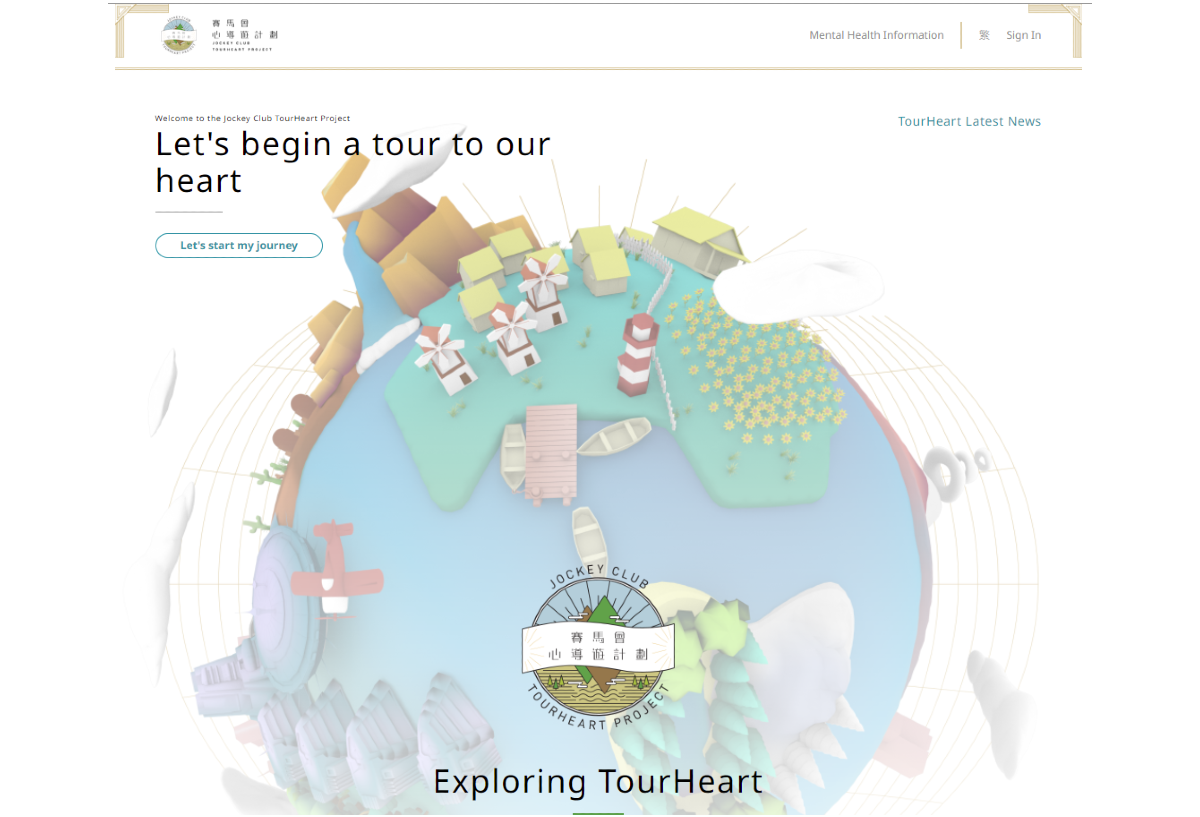
Screenshot of the landing page for the TourHeart platform.

**Figure 4:**
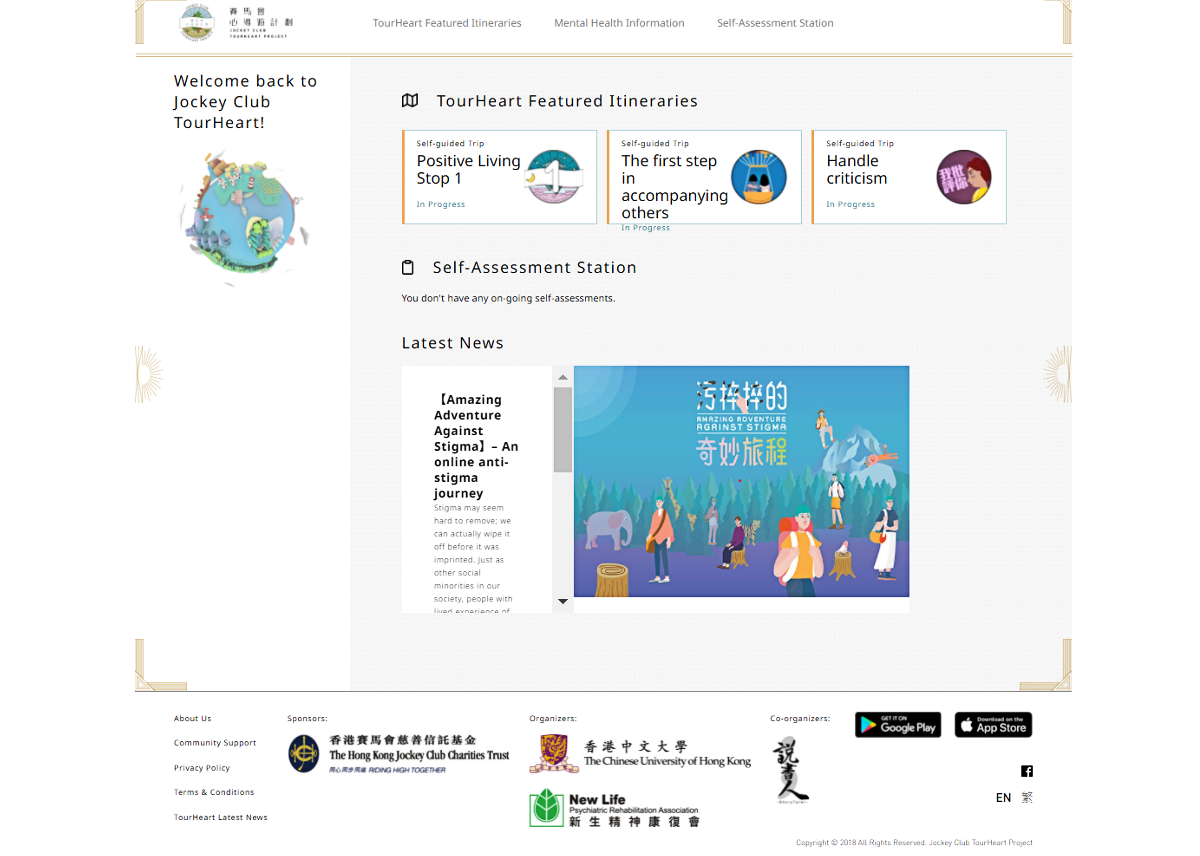
Screenshot of a user’s personalized front page upon signing in to the TourHeart platform.

The platform adopted a minimalist design with pastel tone colors (Figures 3 and 4). A total of 38% (8/21) of the users commented the website design to be attractive and “very eye pleasing” in terms of its choice of colors and the overall layout. Users generally preferred high graphic-to-text ratio. Specifically, the use of multimedia to supplement course content was mentioned frequently (6/21, 29% users). The type of media included animation, videos, voice narration, infographics, and minigames that could facilitate their understanding of concepts introduced in the courses, such as the topic on *thinking traps* that was introduced in the RFCBT course. Users cited the following:

People these days are generally very busy and those who visit the platform are not in the best of mood. Therefore, the platform could use a bit more videos or animations to illustrate their points. No one wants to read a whole page filled with words.

Connie, level 3

Voice narration is good, or at least more illustrations...I don’t like to read as I am already constantly tired....

Ivy, level 3

##### Adequacy

All service users were asked about the technical adequacy of the platform (21/21, 100%); most service users had either positive or no comments about the technical adequacy of the platform (14/21, 67%). The remaining 33% (7/21) of the users commented on various technical inadequacies of the platform. In general, critical comments pertained to ease of navigation, reliability, bookmarking function, and interactivity. Of these 7 users, 5 (71%) users had difficulty with the bookmarking function. A loss of bookmark was experienced after logging out from their sessions. For instance, courses did not resume from points where they were stopped and the users had to restart at different points. Similarly, users reported the audio being switched off when the phone’s screen was in sleep mode.

Moreover, some users were not entirely accustomed to the self-guided nature of the platform. On the TourHeart platform, users were free to choose courses that were made accessible to them at their level of mental health states, such as mindfulness-based training and RFCBT, but some users thought the provision of options was confusing:

I felt confused at the beginning. When I started using the platform, two courses were made available to me, one was mindfulness-related and the other was CBT-related. I remember thinking...which course should I take? Both were available but I felt confused as to where and how I should start.

Kennis, level 3

In terms of suggestions, 10% (2/21) of the users expressed that they would be interested in functions where courses could have different versions of varying lengths as an option for users to choose from for more flexibility, and another 10% (2/21) of the users stated that they would like to have an additional feature where course contents could be archived for offline access. Finally, 5% (1/21) of the users suggested the use of pop-up messages after signing in to draw attention to timely topics.

During the interviews with service providers and project staff (14/35, 40%), comments were primarily about the limitations of the platform back end to properly manage user data, such as limitations in searching and filtering. These comments were grouped into three categories: (1) issues related to user data management (14/14, 100%), (2) data input burden as a deterrent to adherence (4/14, 29%), and (3) limited human resources available owing to the high level of demand for coaching (2/14, 14%). Illustrative quotes pertaining to these 3 categories, respectively, are listed in the following sections.

##### User Management

Service providers and project staff reported a few platform functions that are not user-friendly or missing functions that may expedite and streamline their work in user management.

We had difficulty using the search function to filter out users that fit under specific categories. Quite often we had to use their email that they used for registration; but a lot of users have more than one emails and sometimes we cannot locate a user within the system. The system can be improved with a more sophisticated filtering function.

We had to type in progress notes manually, with no preset categories. For example, age of onset, current medications. This makes progress tracking somewhat difficult. So, we had to set up our own templates using another application.

As part of the intervention, we had to make progress notes for each user after each session. The problem with this function is that it cannot be edited after I clicked submitted. Therefore, if I made a typo or if I had amendment that I want to make to the old progress note, I had no choice but to submit a second one.

The single biggest difficulty was that we spent a lot of our working hours sending out email reminders to users when we could put our time into other uses. We had to send emails manually to guide users every step of the way. For example, welcoming emails to those who newly registered; reminders to those who have not visited the platform for a certain period of time; or emails of encouragement to those who have completed a certain milestone. Indeed, we had to manually create excel sheets to keep track of movements of all the users in the platform.

Since we need to send out so many emails manually, we are left with little time. So, we had to use templates when we gave feedback to the users. If we had more time, we could have done more and done better.

##### Data Input Burden

Service providers and project staff also noted that some users may be turned off by the need to respond to assessment questions prior to starting a course, which may explain user attrition.

From the backend, we could see that users terminated themselves without beginning of a course. We guess they might be scared off by the long assessment questions (24 questions) that they were required to answer before being assigned to the course(s).

##### Content

Another prominent subtheme that arose from the interview discussions was about the courses offered and whether they were useful for the participants in managing their psychological distress. Only participants who received services from levels 2 and 3 (16/21, 76%) commented on this topic. As discussion of the specific components of the courses and whether they were useful in alleviating users’ distress is beyond the scope of this paper, we collated comments that illustrated the general quality of the contents, namely (1) usefulness of contents (16/16, 100%), (2) conciseness (6/16, 38%), and (3) presentation clarity (4/16, 25%).

The contents offered on the platform were generally well received for their effectiveness in facilitating users to deal with emotional vicissitudes for themselves and others. For instance, users said the following:

I have learned to take care of myself emotionally, and through this, take care of my better half, and my family. This is the inspiration I have.

Edward, level 3

I used to have ruminative thoughts, especially after a bad day at work. Now, instead of ruminating about the negative experiences at work, I learned to be mindful when I walk home. This helped me to feel more settled.

Ivy, level 3

Regarding conciseness of the content, users seemed to prefer to have fewer words or even organize the contents into shorter sections or even point forms:

I think they could break down the contents into point forms, as some people might go through the website as if it is a tool book.

Joyce, level 2

I wanted to capture the feedbacks from coach; however, they seemed to be too long and it takes time for me to revisit all of the materials. I would prefer to read the materials in point-form....

Phillip, level 3

Regarding presentation clarity, participants commented that the contents were at appropriate level of difficulty and presented in a way that can be easily understood:

I first learned of mindfulness from the platform. I also read books about mindfulness and found that the information and audio tracks contained are adequate for my daily usage.

Yan, level 2

#### Theme 2: Platform Use Motivators

##### Overview

This part of the study involved 59% (16/27) of the users (level 2: 8/16, 50% and level 3: 8/16, 50%). An open-ended question was asked to solicit participants’ motivations for using the platform. The responses are divided into a few categories in the order of frequency; they include (1) design and navigation (6/16, 38%), (2) the nature of self-help (5/16, 31%), (3) usefulness of contents (4/16, 25%), and (4) free of charge (1/16, 6%).

##### Design and Navigation

Despite encountering challenges in using the platform, the convenience of the web-based platform with its pleasant design was the most frequently mentioned motivating factor that attracted users:

The webpage looks young and vibrant. Secondly, the use of graphics makes it less dull to look at. The design is great; I remember the animated earth on the sign-in page, it gave me a strong impression. I like it.

Rosemary, level 3

It is very convenient, there is no need for any pen and paper.

Joyce, level 2

##### Nature of Self-help

The autonomy and anonymity associated with web-based self-help were considered to be the second most significant motivating factor in platform use. Some of the comments include the following:

The best thing...everything is under my control. It is self-help and the self-assessment enabled me to learn about my current state. It is good.

Priscilla, level 2

I can log-in any time I like, without having to arrange any appointment.

Ivy, level 3

I like the concept of self-help; it is amongst the first of its kind that is available in Hong Kong.

Sunny, level 3

I really enjoyed using it in my own private time. It is less embarrassing compared to in-person interventions. I try to avoid meeting real people for therapies.

Cecilia, level 2

##### Usefulness of Content

Perceived usefulness and satisfaction with the contents offered were the third most frequently mentioned factors that affected platform use. The platform was said to offer new perspectives and information that are helpful to users’ mental well-being:

I find the well-being tips to be useful. I remember one that asked us to smile even though we were not in a good mood, contrary to my expectations, the tip actually worked for me.

Irene, level 2

I feel calm just by looking at the materials.

Cecilia, level 2

The contents offered here are very rich, with audios and teachings about mindfulness...body scan has been particularly helpful for me when I am having sleepless nights.

Joyce, level 2

The part about worries was particularly useful for me. Given the current situation in Hong Kong, local news made me feel distressed and helpless. So, I applied what I learned from the course – I approach or challenge my worries systematically, by asking myself: are my worries constructive? Is there anything I can do to alleviate or distract myself from it? I also learn to communicate my worries with others as now I understand that worrying is normal.

Sunny, level 3

#### Theme 3: Human Coaching

##### Overview

Totally, 30% (8/27; level 3) of users provided feedback about the coaching service that they received as part of their intervention at level 3. All the responses from participants were positive about the recovery-oriented messages they received. Some of them expressed the need for more human interactions in support of using the web-based self-help platform.

##### Recovery-Oriented Aspects

All the users were satisfied with the quality of coaching received through emails. The empowering, person-centered, and recovery-oriented language used by the coaches in the email communications were viewed as “thoughtful,” which also gave them “a sense of companionship.” Totally, 25% (2/8) of the users said that the coaching component was the primary motivator for platform use:

I was impressed with the long and detailed replies from the coaches. I can see that they have poured in a lot of effort to give me feedback and address my concerns. The interactivity of coaching I received from this platform is different from other websites where I mostly accessed just to get information. The collaborative nature of this platform is very useful for me.

Rosemary, level 3

I know that they have read all of the things I have written and provided responses tailored to my needs and worries...the coaches are like my companion.

Kennis, level 3

##### Need for More Human Interactions

When we asked these 8 users for suggestions to improve the coaching service, 5 (63%) of them preferred to have more interactions with the coaches in a timely manner. Of this 63% (5/8) of the users, 60% (3/5) users preferred to have instant messaging with a human coach, 20% (1/5) preferred to have regular phone conversations, and the remaining 20% (1/5) preferred regular face-to-face meetings with coaches.

### Offline Services

#### Overview

This part of the study involved 43% (6/14; level 1) users and 57% (8/14; event organizers) service providers. Data from the semistructured interviews were analyzed to identify topics that were mentioned frequently by the participants. The topics stated by both users and providers who received and delivered level-1 (offline) services, respectively, were collated under 1 general theme: *factors that influence users’* *experience* of activities that were part of the promotion and prevention services. Topics under each theme are reported according to their frequencies.

#### Theme 4: Factors That Influence Users’ Experience of Offline Service

##### Administrative Challenges

This part of the study involved 50% (6/12; level 1) users and 50% (6/12) volunteers. Our data indicated that venues should be easily accessible to the public and properly equipped for comfort (6/12, 50%). As most people in Hong Kong do not own a car, the location of the venue must be convenient and close to a subway station (Mass Transit Railway). In addition, air-conditioned venues should be chosen for events held in the summer, as participants from the public exhibition indicated that they were not able to stay and explore the entire exhibition (held indoor without air-conditioning) owing to the summer heat.

Furthermore, 25% (3/12) of the participants mentioned that an effective registration system was crucial. Features such as immediate notification of registration outcome, update of waitlist status, and reminder before the event using instant messaging were mentioned. These features were said to be particularly important for people with busy schedules.

Among event organizers, a few (4/6, 67%) mentioned that the biggest administrative challenge was working together as a team because they all come from diverse professional backgrounds. All of these volunteers (4/4, 100%) suggested regular team-building exercises to foster better work relationships.

##### Perceived Impact

This part of the study involved 65% (11/17) users (level 1: 6/11, 55% and level 4: 5/11, 45%) and 35% (6/17) volunteers. Most users and providers (10/17, 59%) gave positive comments about the public mental health promotion events centered on the theme *accompany*. These workshops highlighted the core concept of how everyone can be both the supporter and recipient of support, which reduces stigma of help seeking. During these events, participants were also equipped with skills to identify the more obvious signs of depression and anxiety to promote awareness within themselves and among others. In general, participants found all the events to be very “down-to-earth” (Jasmine, level 1) and the concepts to be easy for laypeople to grasp. The intended messages of the psychoeducational workshop and exhibition were also said to be delivered effectively and thoughtfully (10/17, 59%). For instance, the event organizers, some of whom worked closely with people with lived experience to deliver talks or *storytelling sessions,* found that sharing of lived experience within small groups was particularly impactful in building connections and strengthening empathy between people with lived experience and the audience. In addition, in the public exhibition, participants were invited to inflate balloons to reveal messages printed on them. During the process, participants had to exert patience and effort, similar to the qualities required to be good listeners, which was the key message of that exhibition. The psychoeducation talks were also said to “leave a lasting impression” (Ivy, level 1) that “normalizes mental illnesses” (Harry, level 1) without overwhelming lay participants. However, at the same time, some participants also expressed a wish to receive more in-depth knowledge or skills-based training. For example, some suggested that the organizer conduct a series of workshops progressing through the details of various mental health–related content so that participants can acquire the full range of skills on various aspects of mental health management.

Regarding level 4, users commented on LiCBT (face-to-face or via videoconferencing). Of the 5 level-4 users, 1 (20%) user decided to drop out of the service as she preferred talk therapy such as counseling. The rest of the users (4/5, 80%) reported positive experience with the service, including feeling a sense of warmth and rapport with the psychological well-being officer (5/5, 100%) and finding the guidebooks used in LiCBT as useful (3/5, 60%). Notably, the presence and interactions between the psychological well-being officer and user were mentioned by all users as the most positive aspect of level-4 service, marking the human element indispensable in both our web-based and offline services:

It’s the sense of being understood...techniques aside...when I talk to the PWO, I felt that she was present and was willing to listen...that already made me feel so much better.

Eugene, level 4

## Discussion

### Principal Findings

Overall, our findings highlighted the importance of person-centered design that emphasizes autonomy and competence, with relevant information empowering users to self-help. The implementation of a web-based stratified stepped care approach to mental health support is driven by the need for increased accessibility and improved availability of relevant mental health services to the general working population. Although studies in the field have demonstrated the effectiveness of digital mental health interventions [9-11], their uptake is still moderately suboptimal [31]. Moreover, an integrated web-based platform curating relevant mental health tools and interventions for promotion, prevention, and treatment of common mental health conditions is lacking. With the recent development of web-based psychotherapies that use artificial intelligence and chatbots, novel technology-based therapeutic experiences with better user experience are being increasingly recognized.

This qualitative study was designed to evaluate a stratified stepped care platform that includes both offline and web-based mental health services. Primarily, the desired and positive qualities of the TourHeart platform were explored from the perspectives of service users and providers. Factors that were identified to be important in improving users’ experience in offline services include administrative challenges and perceived impact. In the evaluation of web-based components of the platform, 3 broad themes emerged from the interview data: quality of the platform, drivers for platform use, and human coaching. Across the themes, a visually attractive platform with high graphic-to-text ratio and multimedia content, seamless user experience in navigation with a sophisticated bookmarking function that can cater to users who may be using the services in between their schedules and thus might log off intermittently, and concise down-to-earth presentation of relevant content emerged as the most important. These qualities are consistent with previous research that shows platform’s esthetics to be facilitative of initial uptake [32,33]. Thus, these are critical design parameters that developers should focus on while developing future web-based platforms. In addition to esthetics and ease of use, users also preferred the sense of autonomy and anonymity that is associated with the web-based platform [34,35] along with alleviation of distress that drives their continued use. In particular, many people may regard using a web-based platform as less embarrassing and more preferred than conventional face-to-face therapies.

Although human interactions via email coaching (level 3) and via face-to-face or videoconferencing with psychological well-being officers (level 4) are considered to be indispensable components of the platform, some contradictions were noted. Specifically, although self-help and the resulting sense of autonomy and mastery were found to improve overall use, several participants found self-directed navigation of low-intensity psychological interventions to be challenging, as they had to pace and tailor the materials by themselves without support from other people. Another contradictory finding was the need for human interactions to support platform use. These contradictory findings are broadly consistent with the findings from previous meta-synthesis [36] that proposed 2 critical system characteristics to address the competing needs of users. The first characteristic was collaboration between users and the system. This sense of collaboration should be different from having a sense of *enforced autonomy,* where users are burdened with having to navigate the system without any type of external support. The second characteristic was connection that can be achieved by balancing the need for personal privacy with increased distance and maximizing interactions with therapists or web-based coaches through messaging, forum discussions, emails, and so on.

In summary, findings of our study are consistent with existing literature and design standards for digital mental health services [37,38] and highlight the importance of services to be both user-centric and comprehensible to all users. In addition to these front-end features, a back-end system that is effective for system administrators to manage user data is also essential for effective service delivery, including functions for searching, filtering, and reporting. Notably, themes generated from our study were found to converge with the objective domains covered in the Mobile Application Rating Scale [39], namely, users’ engagement, functionality, esthetics, and information quality, which supports the validity of our findings.

Regarding future directions, findings of this study suggested that developers should integrate more motivational and interactive components to improve users’ engagement, such as statements of encouragement and support, open-ended and fill-in-the-blank entries for users to reflect on the related questions and apply course exercises to themselves in their daily lives. In addition to improving initial uptake, the general esthetics of the web-based platform can also facilitate users’ adherence [32,33]. Moreover, clear and concise information is needed to provide a seamless self-help experience for users. Our data indicated that a lack of understanding about the stratified stepped care service model might render the user experience moderately unclear. A lack of road map in guiding the course of treatment and manual data input were 2 factors that hampered users’ overall use. Therefore, service expectations of users should be taken into account in the design phase to achieve better alignment. Owing to the dynamic nature of stepped care service where users are subjected to stepping up and down to interventions that vary in intensity and interactivity in accordance with their self-reported mental health status, changes to the types or length of interventions could be confusing to some users. In fact, evidence from another qualitative study conducted by the authors of this paper (EWS Tsoi, PhD, unpublished data, 2021) indicated that some users are ambivalent to this approach to care. Specifically, several users with high levels of distress were unwilling to be stepped up and receive face-to-face care. These participants preferred to continue with web-based care to maintain their anonymity. The fear of disclosure and feelings of embarrassment during face-to-face therapies might deter some individuals from stepping up to face-to-face interventions. Future web-based services should consider tailoring to the needs of this particular group of users using blended web-based–offline approach or web-based psychological interventions that are suitable for people with more severe levels of anxiety or depression.

Although technical struggles were not the focus of our study, the difficulties encountered by our participants and their preferences for support in platform use suggested that support function that is instant and highly accessible should be included. In recent years, text-based conversational agents (eg, chatbot) have emerged to provide timely and reliable customer service experience in fields such as banking and web-based shopping [40,41]. Chatbots have also been increasingly adopted in health and mental health care settings [42-44]. Although its use and effectiveness are still largely experimental, how this new technology could be leveraged is a productive area to explore. Ideally, the involvement of real persons to provide support behind the screen should be kept to a minimum so that services can be scalable and delivered more cost-effectively to more users [45]. Future studies can further explore ways in which paraprofessionals and peer support workers can deliver timely support remotely.

TourHeart is operated under the stratified stepped care model that is similar to Stepped Care 2.0, which focuses more on user experience and recovery in a progressive manner [46]. Furthermore, TourHeart covers the spectrum of mental health needs, which includes mental health promotion, stigma reduction, mental illness prevention, and treatment for common mental disorders. The second phase of TourHeart (Jockey Club TourHeart+ Project) models on Stepped Care 2.0 by using a person-centered approach and machine learning, where users can choose options at different levels and receive regular feedback and reports on growth or deterioration so that they can retain a sense of autonomy. Iterative user experience research was also conducted during the course of platform development and maintenance. By understanding the perspectives of various stakeholders, web-based mental health platforms may be better designed in the future to provide timely and bespoke web-based and offline mental health–related information, skills, and interventions to users, with technical support and consideration of user experience to improve the acceptability and effectiveness of digital mental health services.

### Strengths and Limitations

Using a bottom-up analytical approach, this study has identified salient aspects of the web-based self-help platform, TourHeart, that were important to both users and service providers and were the main drivers of platform use. In addition, our findings highlighted the importance of balancing our need for support and need for privacy and autonomy, which could potentially be addressed through enhanced system collaboration and connections, which were explained in the previous section. Factors that promote engagement can also be further explored through user experience research.

This study was limited by the heterogeneity of the sample in terms of age, gender, and clinical representation. Participants who agreed to participate in the study may also pose a risk of self-selection bias. In addition, comments on the platform in general were invited and participants without in-depth knowledge of user experience and design may not be able to conceptualize what an *ideal* platform should be; hence, the desired features of inadequacies pointed out should be interpreted with some caution.

### Conclusions

This study identified a few issues that need to be addressed to enhance the adoption and usability of web-based mental health platforms. These insights were incorporated into the development of phase 2 of TourHeart (TourHeart+). Given that people’s needs and preferences for mental health services may evolve over time, incorporation of iterative and rigorous user experience research into the development and maintenance of web-based mental health self-help platforms is urgently needed.

## Abbreviations

CBT: cognitive behavioral therapy
LiCBT: low-intensity cognitive behavioral therapy
RE-AIM: Reach, Effectiveness, Adoption, Implementation, and Maintenance
RFCBT: rumination-focused cognitive behavioral therapy
